# Towards an Integrative Theory of Bullying in Residential Care for Youth

**DOI:** 10.3390/ijerph19095166

**Published:** 2022-04-24

**Authors:** Ivana Sekol, David P. Farrington, Jane L. Ireland

**Affiliations:** 1 Department of Criminology and Social Sciences, University of Derby, Derby DE1 1DZ, UK; 2 Institute of Criminology, University of Cambridge, Cambridge CB3 9DA, UK; dpf1@cam.ac.uk; 3 Ashworth Research Centre, School of Psychology and Computer Science, University of Central Lancashire, Preston PR1 2HE, UK; jlireland1@uclan.ac.uk

**Keywords:** theory, bullying, victimization, residential care, out-of-home care, adolescents, youth

## Abstract

To date, no theory of bullying in residential care for youth has been proposed. By drawing on the results of the existing research on bullying and peer violence in youth residential care and adapting the Multifactor Model of Bullying in Secure Settings (MMBSS), this paper proposes the first integrative theory of bullying in residential care—the Multifactor Model of Bullying in Residential Settings (MMB-RS). The paper first summarises the existing empirical findings on bullying and peer violence in residential care for youth and describes the MMBSS. It then moves on to proposing and describing the MMB-RS. In a nutshell, the MMB-RS assumes that bullying in residential care is shaped by a dynamic interaction between a complex set of individual and contextual factors. The model also takes into account the *interaction between bullies and victims*, thus explicitly considering the social interactional components of bullying and victimisation and offering possible explanations of the sizable overlap between bullying and victimisation in residential care, including the possible contributions of residential peer cultures. The paper concludes by noting the importance of empirically testing the MMB-RS and proposing a programme of research that may be helpful in testing it.

## 1. Introduction

The prevalence of bullying amongst young people in residential care is significantly higher than amongst children in schools (for a comparative review, see [1]). Prevalence is clearly informed by adopted definitions, with bullying commonly defined as direct or indirect aggressive behaviour, which is repeated over time, and includes a power imbalance [2]. Although Olweus’s [2] definition also includes the intention to cause harm to victims, it has been argued that in closed environments some bullying may well be unintentional [3,4]. The consequences of bullying may be more profound and long-lasting for residential care victims, due to such facilities being relatively inescapable social environments that often care for young people with disadvantaged and traumatic life histories, challenging behaviour and emotional problems [5,6,7,8]. This notwithstanding, compared to research on bullying in schools, research on bullying amongst young people in residential care remains relatively scarce. Early residential care bullying research was mainly descriptive and/or qualitative, predominantly reporting on the prevalence of bullies and victims and types of bullying [4,5,9]. 

Over the last decade, however, academic interest in this topic has been steadily increasing worldwide, with noteworthy efforts to: (a) quantitively establish individual, environmental and social predictors of bullying in residential care for youth [7,10,11,12]; and (b) qualitatively explain the processes underlying peer violence in youth care [13]. The results of these research efforts have suggested that bullying in residential care is likely to arise from the interaction between bullies, victims and other residents in residential environments, which provide opportunities for bullying [14]. Nevertheless, apart from one attempt (e.g., 7; for details see below) to test one discrete element of Ireland’s [15] Multifactor Model of Bullying in Secure Settings (MMBSS hereafter), no other residential care research has tested a theory. More importantly, no attempts have been made to develop a comprehensive theory of bullying in residential care that incorporates and links the results of the existing residential care bullying research. 

The need for a comprehensive theory is important since prevention methods should be based on well-developed theories. The reality in practice, however, is that many methods are based on disconnected results of previous research and inconsistent hypotheses [14]. It is, therefore, important to develop an all-embracing theory of bullying in residential care to guide future prevention and research efforts. It is equally important to test any proposed theory, ideally through a programme of intervention research. Based on a summary of the results of previous residential care bullying and peer violence research and the MMBSS developed by Ireland [15], this paper is the first to propose a theory of bullying in residential care, as well as a programme of intervention research in which the proposed theory can be tested. The paper starts with a review of four main categories of results of all published residential care bullying and peer violence research to date: (1) the nature and extent of bullying in residential care; (2) personal characteristics of residential care bullies and victims; (3) the large overlap between bullying and victimisation in residential care and characteristics of the “bully/victim” group; and (4) the context of residential care bullying, namely the physical and social residential care environment. This paper then moves on to describing the MMBSS [15], which is a more suitable basis for theorising about bullying in residential care than theories of bullying amongst schoolchildren. Finally, an integrative theory of bullying in residential care, an adaptation of the MMBSS, is proposed and a programme of intervention research suitable for testing the adapted MMBSS theory is put forward.

### 1.1. Review of Previous Residential Care Research on Bullying and Peer Violence

Although the main aim of this paper is to propose a theory of bullying in residential care, this paper also reviews research on peer violence in residential care in order to offer an empirical basis. Importantly, the main difference between bullying and peer violence is in the frequency of occurrence, with incidents of direct or indirect aggression considered bullying if they occur repeatedly (i.e., two or three times a month or more often; [2]), whereas peer violence usually refers to more sporadic or one-off incidents of direct or indirect aggression. The majority of the papers reviewed next have collected data on bullying, not peer violence, usually using anonymous self-report questionnaires that list behaviours indicative of direct and indirect bullying (without using a definition of bullying). Specific behaviours are listed in such questionnaires to avoid different interpretations of the term bullying by residents, which has proved to be particularly useful for non-English speaking participants. However, since it has been proposed that, in closed social environments such as prisons, the fear of future victimisation could be more important in defining bullying than the repetition of aggressive acts [3], the most important research on peer violence in residential care is also captured. 

### 1.2. The Nature and Extent of Bullying in Residential Care and Staff Awareness of the Problem 

The prevalence of bullying and peer violence in youth residential care is considerably higher than in schools. For instance, in their qualitative study of the context of peer violence in children’s homes, Barter et al. [5] interviewed 71 residents aged 8–17 from 14 children’s homes in England and found that almost all had experienced verbal attacks, either as victims or perpetrators. Over 85% of residents reported being victims or perpetrators of physical violence, while nearly 50% of residents reported being victims or perpetrators of “physical non-contact violence” (e.g., property attacks). Although Barter et al. [5] did not necessarily study repeated incidents of violence (i.e., bullying), most residents described the above types of peer violence as having an enduring negative emotional impact on their lives. 

In their study of the overall experience of living in care amongst 223 young people from 48 English children’s homes, Sinclair and Gibbs [9] found that over 40% of residents were bullied. Their results were based on a fairly broad definition of bullying that was provided in interviews with residents and included physical violence, threats and systematic humiliation, as well as other experiences, which were likely to cause distress to victims. Despite the broad definition, this study failed to investigate different forms of bullying and focused more broadly on experiences of residential care. It also remained unclear over which time period bullying was measured and whether only repeated incidents were considered bullying. 

Employing an anonymous self-report questionnaire listing behaviours indicative of bullying and victimisation in a national sample of 601 residents of Croatian children’s homes and correctional homes, aged 11–21, Sekol and Farrington [4] found that more than 70% of residents in both correctional homes and children’s homes were involved in bullying two or three times a month or more often, either as bullies or victims. No significant differences were found in the prevalence of self-reported bullying occurring in children’s homes versus correctional homes, suggesting that bullying in care may be predominantly determined by residential peer cultures and other institutional variables, rather than by the psychological makeup of the residents referred to in the two types of facilities. Less direct forms of bullying, such as gossiping, spreading rumours, stealing or damaging someone’s belongings, were roughly equally prevalent as more direct forms of bullying (e.g., physical) in both types of facilities. 

In both facilities, bullying usually occurred in bedrooms during the night, considered a potential likely result of decreased supervision of residents during that time. However, a lot of bullying in both types of facilities was taking place in living rooms, yards and corridors, suggesting that staff either did not supervise those areas appropriately or staff presence was failing to deter bullying. Relatedly, about 50% of victims noted that they never reported their victimisation to staff. Around half of residents believed that staff rarely or never knew about bullying, while around a quarter believed that staff rarely or never tried to stop bullying when they knew about it. Indeed, the data collected from 140 residential care staff at the same time and in the same facilities in which the data was collected from the residents, demonstrated how, compared to residents’ self-reports, staff significantly underreported the overall prevalence of bullying and victimisation in their facilities. Although staff were more aware of the prevalence of some types of bullying than of other types, they had difficulties identifying the accurate times and places of bullying and held stereotypical views about victims and bullies. Staff also reported using reactive rather than proactive anti-bullying strategies (for details, see [1]). 

Attar-Schwartz [16], Attar-Schwartz and Khoury-Kassabri [10], and Khoury-Kassabri and Attar-Schwartz [17] obtained data on physical, sexual, verbal and indirect bullying using questionnaires containing a list of behaviours indicative of these types of bullying from 1324 Jewish and Arab participants aged 11–19 years from 32 residential care facilities in Israel. The results demonstrated that 73% of residents were verbally bullied at least once in the previous month, while 62% and 56% of residents were bullied indirectly and physically, respectively. Around 40% reported being the victim of at least one act of sexual bullying in the month prior to the survey, with the rates of sexual bullying being similar for girls and boys.

Using a self-report questionnaire that listed various behaviours indicative of bullying, Wright [18] compared the rates of bullying and victimisation amongst 50 male adolescents in residential care in the Southern USA to the rates of bullying and victimisation amongst 50 male adolescents in public schools. Although her study did not report on the exact rates of bullying and victimisation, Wright [18] found that males in residential care reported significantly higher levels of bullying and victimisation than the comparison group in schools. In a similar study that used a questionnaire measuring bullying and victimisation and that was conducted with 1481 school children and 56 children from residential care in Spain (both groups aged 10–15), Yubero, Navarro, Maldonado, Gutiérrez-Zornoza, Elche and Larrañaga [19] also found that young people in residential care reported significantly more bullying and victimisation than comparison group counterparts. 

In their qualitative interviews and/or focus groups with 123 residents from residential care facilities in Bulgaria, France, Greece, Italy and Romania, Mazzone, Nocentini and Menesini [20] found that all residents stated that bullying was very common in their facilities. However, these results need to be interpreted with caution, given very small sample sizes in some countries (i.e., n = 17) and the use of Olweus’s definition of bullying, which many residents reported not to understand due to challenges in translating the term “bullying”.

Conclusions thus far from the research indicate strong evidence of bullying representing a concern for young people placed in residential care. The forms of bullying are varied, there appears some similarity across gender, with staff perceptions of bullying appearing considerably disparate from the self-report of residents. Thus, the basis for considering bullying as a problem has been met and the next area of consideration is one of who is involved in these abusive interactions.

### 1.3. Personal Characteristics of Residential Care Bullies and Victims

While Barter et al. [5] and Sinclair and Gibbs [9] found that bullying was predominantly carried out by older residents, in their quantitative self-reported survey in Croatia, Sekol and Farrington [11] found that neither male nor female bullies were older than other residents. However, in Sekol and Farrington’s [11] study these results only completely held for female bullies. Amongst males, such a result was influenced by the very young age of “bully/victims” who were included in the sample of bullies. When only male “pure bullies” were looked at, they were significantly older than other residents. Sekol and Farrington [11] further found that both male and female bullies were neurotic, careless, disagreeable, likely to bully others in school and likely to hold attitudes supportive of bullying. Male bullies also lacked affective empathy and were extraverted. They tended to bully others in their previous care facilities and were more likely than other residents to be placed in care for problematic behaviour, suggesting that bullying may be persistent amongst male residents.

In terms of personal characteristics of residential care victims of bullying, both Sinclair and Gibbs [9] and Attar-Schwartz and Khoury-Kassabri [10] found that residential care victims were younger than other residents, while in Sekol and Farrington’s [12] study this held only for male victims. However, Sekol and Farrington [12] found that both male and female victims were neurotic, had low self-esteem and tended to think that bullying was a normal part of life in residential care, thus reflecting a fatalistic acceptance of worryingly high levels of bullying in Croatian residential institutions. Female victims were also disagreeable and lacking in conscientiousness, while male victims were victimised during their previous placement, at the beginning of their current placement and in school, thus demonstrating continuity in victimisation. The stability of victimisation over time was also evidenced by Sinclair and Gibbs [9], who found that more than 50% of residents who were bullied in their previous facilities were also bullied in their current children’s home, while this held for only around a third of residents who were not bullied in their previous facilities. While bullying behaviour seems to occur more often amongst residents who were admitted to care, because of their troublesome behaviour, Sinclair and Gibbs [9] found that residential care victims were less likely than other residents to be admitted to residential care because of their problematic behaviour. 

Attar-Schwartz and Khoury-Kassabri [10] found that residents who perceived themselves as having low self-efficacy were more likely than other residents to be victims of indirect bullying. Similarly, residents with adjustment difficulties were more likely than other residents to be victims of verbal, indirect and sexual bullying [10,16].

### 1.4. The Overlap between Bullying and Victimisation in Residential Care and Characteristics of the “Bully/Victim” Group

Children who are both bullies and victims have been consistently found in research on bullying in schools [21,22,23,24,25,26], but their prevalence in schools has typically been considerably lower than the prevalence of “pure victim” and “pure bully” groups. However, the opposite has been reported in studies conducted amongst young offenders, where the prevalence of the “bully/victim” group has been typically much higher than the prevalence of “pure bullies” or “pure victims” [3,27,28,29,30]. In both schools and young offenders’ institutions, the “bully/victim” group was described as especially problematic and characterised by many externalizing and internalising symptoms [31]. However, these descriptions of “bully/victims” were not rooted in empirical evidence that would demonstrate that the bully/victim group in these studies was qualitatively different [31]. 

In line with the research in young offender institutions, Sekol and Farrington [4] found that 58.8% of all self-reported victims in their above-described national research were also bullies and 75.1% of all bullies were also victims. The bully/victim group was, therefore, the most prevalent group in Croatian residential care, with 37% of residents being classified as “bully/victims”. Twenty-seven percent of residents were classified as “pure” victims, while 11% of residents were “pure bullies” and 25% of residents were not involved in bullying. However, Sekol and Farrington [31] went a step further and examined whether the “bully/victims” found in their 2009 study [4] differed from “pure bullies” and “pure victims” in kind or only in degree, by comparing “bully/victims” to “pure bullies” on their background characteristics, bullying and victimisation histories, the ways they bullied, how they reacted to their victimisation, their attitudes towards bullying, personality traits, empathy and self-esteem. Differences in degree would imply that “bully/victims” manifest the same behaviour or have the same traits as “pure victims” or “pure bullies” but demonstrate slightly more or less of these behaviours/traits. Differences in kind would mean that “bully/victims” have characteristics or manifest behaviours that are not present in either pure bullies or pure victims, or vice versa.

Their results demonstrated that “…similarities between “bully/victims” and either “pure bullies” or “pure victims” far outweighed their differences, demonstrating that bully/victims were not in any way unique” [31], suggesting that differences between “bully/victims” and “pure bullies” and “pure victims” were in degree, rather than in kind. Given that no unique personal characteristic were found for the “bully/victim” group, the authors suggested that the high overlap between bullying and victimisation in residential care may have been caused by a dynamic interaction between bullies and victims in the special context of the residential social and physical environment, rather than by residents’ personal characteristics. It was also suggested that, when it comes to their psychological makeup, “bully/victims” in residential care may simply be bullies or victims for whom a “bully/victim” status may have been a short-lived, temporary experience influenced by the immediate situation [31]. That “bully/victims” may indeed not be a qualitatively unique group was also confirmed in the above-described follow-up study in residential care [7], where “bully/victims” were compared to “pure bullies” and “pure victims” on several variables measuring their perceptions of the social and physical residential care environment. Collectively, this directs to the role of the environment as a notable element, highlighting the importance of capturing the context within which bullying is taking place.

### 1.5. The Context of Residential Care Bullying and Peer Violence: The Physical and Social Residential Environment 

In their qualitative study in children’s homes, Barter et al. [5] identified six institutional factors that were related to peer violence amongst residents: (1) the inconsistent use of (or lack of) anti-violence policies and procedures; (2) different interpretations of the Children Act [32] by staff; (3) a lack of opportunities for residents to share their experiences and thoughts about peer violence in regular residents’ meetings; (4) inadequate referrals; (5) large home size and poor furnishing of the facility; and (6) a poor staff to children ratio. 

Barter et al. [5] also found that residents had their own residential peer culture, which was shaped by residents’ own rules and hierarchical peer dynamics, in which dominant residents or “top dogs” often used control, coercion and violence to dominate their peers. In such hierarchical peer groups, residents usually perceived that admissions of new residents threatened their own places in the group hierarchy. To protect their positions in the group, residents tended to rely on bullying. This ranged from physical aggression to “initiation ceremonies”, which were used to “test out” new residents. If new residents did not defend themselves adequately, they were automatically considered weak and positioned at the bottom of the hierarchy. Overall, violence was normalised amongst residents and both residents and staff perceived peer hierarchies as normal, with staff noting that sometimes they used residents’ “pecking orders” for establishing and maintaining control in the facility. 

In her qualitative research based on focus groups conducted with 120 residents, aged 11–21, from 20 Croatian care institutions, Sekol [13] also found strong residential peer cultures, which “…portrayed a rich and complex residential social world embedded in [residents’] norms, rules and values”. The residents’ value system was centred on friendship and solidarity or, alternatively, the appreciation of material goods (e.g., money, cigarettes, mobile phones, etc.). Friendship was particularly valued because residents felt that, due to their similar life experiences, the support they received from their fellow residents was more credible than staff support. The importance of material goods was based on their general shortage in residential facilities. Such a residential value system was translated into a “residential code”, with explicit rules that shaped residents’ behaviour. The main rules of the residential code were: “do not grass on other residents; do not steal from other residents; do not be stingy [i.e., non-generous]—Share with others; protect each other when there is an external threat (‘one for all, all for one’); help others—Be a friend; do not be haughty; respect older residents; do not make hurtful comments about someone’s family; do not lie; and do not be double faced—Be yourself” [13]. In line with classic prison ethnographies [33], the residential code was predominantly based on prosocial values and norms. However, violations of the residential rules often served as justification for violence amongst residents. Given a high prevalence of both bullying and victimisation in the Sekol and Farrington study from 2009 [4], it appeared that the residential code only represented an ideal, not the actual behaviour of residents, and that residents predominantly did not conform to the code [13,34]. However, if residents conformed to the residential code, they would earn respect from others.

Apart from the residential peer culture, Sekol [13] identified three further themes that contributed to the relationship between living in care and violence amongst residents: (1) vulnerability in early stages of institutionalisation; (2) stigmatisation, frustration and deprivations; and (3) a poor relationship between residents and staff. All four themes, including residential peer cultures, were mutually inter-related. For instance, stigmatisation, deprivations, frustration and a poor relationship with staff all added to creating residential peer cultures, while residential peer cultures further contributed to a poor relationship with staff and victimisation of residents at early stages of their institutionalisation. In line with the findings by Barter et al. [5], deprivations of material goods usually led to theft or intimidation/force to obtain scarce goods, while a poor relationship with staff both contributed to and was caused by staff either ignoring problems between residents or relying on violence between residents as a means of controlling or punishing them. Overall, residents believed that staff viewed bullying as a ‘normal’ part of growing up in care, felt underestimated by staff and often perceived staff decisions as unfair and illegitimate. 

In her follow-up quantitative study, Sekol [7] examined the relationship between residential care bullying and victimisation and the social and physical residential environment amongst 272 residents aged 11–21 from 10 residential care facilities in Croatia. The results demonstrated that bullies and victims, regardless of gender, reported having significantly less peer support than other residents, although a lack of peer support was more pronounced for victims than for bullies. Male bullies were more likely than other residents to report having insufficient staff support, as well as to perceive their facilities as having problems with ventilation, heating, cleanliness and food. Female victims reported having a poor relationship with staff and being dissatisfied with heating, ventilation, furnishing and domestic facilities. 

While some studies [5,9] found that more peer violence occurred in large institutions, others have not replicated such findings [10,17]. However, there is evidence that negative residential peer cultures might be stronger in more secure, closed residential facilities, such as those housing young people with troublesome and/or antisocial behaviour [4]. 

In line with the findings by Sekol [7] presented above, Attar-Schwartz and Khoury-Kassabri [10] found that residents who felt that their residential peer group was not supportive and friendly were more likely than other residents to be victimised verbally and indirectly. Residents who were subjected to physical maltreatment by staff were more likely to be victims of verbal, indirect and sexual bullying [10,16], while residents who perceived residential anti-violence policies as inconsistent, unclear and unfair were also more likely than other residents to be victims of sexual bullying [16]. 

Consequently, there appears a notable association between experiences of bullying and victimisation and the context within which this occurs. This is consistent with the suggestion that aggression does not occur within a vacuum but is instead a product of environmental components and how an individual is interacting with these. This has formed the basis of multifactorial explanations of bullying in secure settings, such as prisons [15].

### 1.6. The Multifactor Model of Bullying in Secure Settings (MMBSS)

While Bronfenbrenner’s [35] ecological model has been used to explain bullying in schools [36,37], the above review of existing research of bullying in residential care demonstrates that both residential care bullying and the residential care environment differ considerably from school bullying and the school environment. Since many elements of the MMBSS have been found to be related to bullying and victimisation in care, it has been suggested that the MMBSS may provide a useful basis for theorising about bullying in residential care [7]. 

The MMBSS [15] represents the only comprehensive theory that attempts to explain bullying in closed social environments. It was a development of the Interactional Model of Prison bullying [3], which considered prison bullying a product of individual characteristics and the environment. However, this was a basic conceptual model that specified the pathways through which bullying could develop. The MMBSS progressed from this, taking advantage of increased research into prison bullying, and it has been applied to management and intervention [38] and underpinned evaluations [39]. According to the MMBSS, prison bullying is a product of an interaction between the prison environment and prisoners’ personal characteristics (for the full MMBSS [15]; for a shorter review [7]). The model describes two pathways to bullying in prisons, both of which are ultimately driven by the environmental context: (1) the “desensitisation” pathway and (2) the “environment and prior characteristic” pathway. 

The “desensitisation pathway” assumes that the prison environment is marked with frequent aggressive incidents, which lead to the normalisation of violence, where prisoners gradually become desensitised to aggression. This route is thought to enhance existing individual characteristics, namely the ‘imported’ factors that individuals bring with them to the environment that are likely to promote aggression and promote aggressive-supportive attitudes. In includes a specific role for acute experienced emotions, such as fear and/or hostility, which raises the potential for aggression and provides a route whereby bullying could occur. Importantly, it recognises an emotional route towards the bully/victim role and a means through which any resulting aggression is reinforced via the social environment. The “environment and prior characteristic” pathway considers the interaction between the prison environment and stable characteristics, arguing for a more trait-driven approach towards the route to becoming a pure bully. It therefore focuses on this role and notes how the environment serves to maximise the manifestation of traits that contribute to aggression, including an equal role for both the social and physical environment. 

The MMBSS makes particular reference to certain aspects of the physical and social environments in prison that are thought to promote these routes. Promoting features of the physical prison environment include large numbers of people detained together within a small space, limited access to material goods, scarce environmental stimulation and a high prisoner to staff ratio, resulting in predictable and limited staff supervision of prisoners. The social prison environment includes the presence of prisoner subcultures with pecking orders, norms and values that promote peer hierarchies, authoritarian relationships between staff and prisoners based on control, negative attitudes towards victims and low genetic and attachment relationships.

## 2. Towards an Integrative Theory of Bullying in Care: The Multifactor Model of Bullying in Residential Settings (MMB-RS)

**Figure 1:**
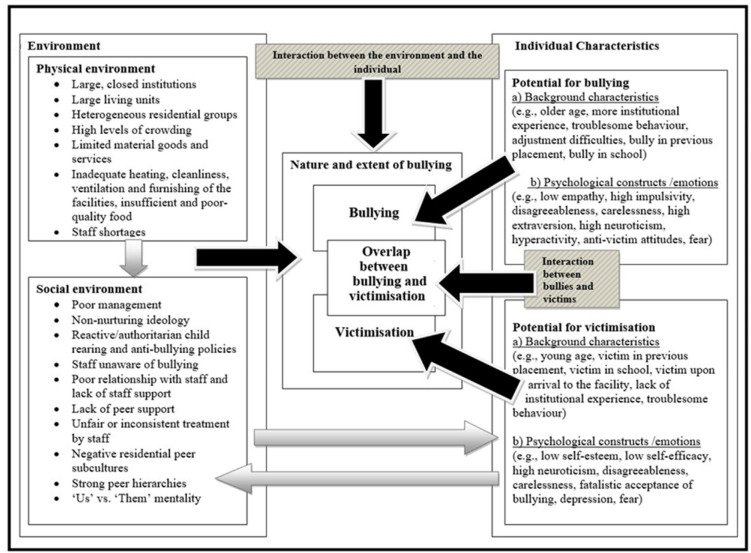
The Multifactor Model of Bullying in Residential Settings (MMB-RS).

By integrating the factors related to bullying and victimisation in residential research, as described previously, the model presented in Figure 1 can be proposed. This model adapts the MMBSS [15] for residential settings. The proposed model—Multifactor Model of Bullying in Residential Settings (MMB-RS hereafter)—assumes that bullying in residential care is also shaped by a complex set of individual and contextual factors that interact dynamically. As marked by black arrows in the Figure, bullying and victimisation, as well as the overlap between the two, is considered the result of: (a) the residential environment; (b) individual characteristics of residents; (c) the interaction between the individual and the environment; and (d) the interaction between bullies and victims.

By taking into account the *interaction between bullies and victims* and including *both of the original MMBSS pathways* in a single pathway, the MMB-RS applies the MMBSS basic components to a residential setting. Not only does such an approach explicitly consider the social interactional components of bullying and victimisation, it also offers possible explanations of the sizable overlap between bullying and victimisation in residential care. The MMB-RS also departs from MMBSS by describing in more detail *interactions between all other elements* included in Figure 1 (see the next two paragraphs for details). As indicated by grey arrows and patterned boxes, the model also makes those interactions more explicit and visible. Furthermore, the MMB-RS includes more *specific individual variables,* making a clear distinction in terms of how these variables differ for bullies and victims (e.g., older age and anti-victim attitudes signify a potential for bullying, whereas younger age and a fatalistic acceptance of bullying signify a potential for victimisation). The model also includes some *new environmental variables* (i.e., the size and composition of residential groups and the ideology and management of the residential placement) and renames certain environmental elements so that they better reflect the residential care setting (e.g., it refers to child rearing techniques rather than hierarchical structures). Finally, although the discussion that follows mainly relies on empirical evidence collected in residential care research and arguments proposed in the MMBSS, it also makes reference to a wider literature concerning the sociology of prison life and ethnographic residential care literature. 

Before moving on to describe the MMB-RS, it is important to note that the elements presented in Figure 1 are separated only for ease of presentation. As noted, the model views bullying as a result of interactions between multifaceted contextual and individual factors. Therefore, multilevel and dynamic interrelationships between virtually all individual and environmental factors should be borne in mind when interpreting the model. Starting points and final destinations of each of the arrows included in Figure 1 should also be taken into account. For instance, the grey arrow leading from environmental factors to individual characteristics arises from the outer ‘environmental box’ and terminates in the outer ‘individual box’, indicating that *both* the physical and social environment will have an influence on already existing individual potentials for both bullying and victimisation. The grey arrow leading from individual characteristics to environmental factors, however, arises from the outer ‘individual box’ but terminates in the ‘social environment box’. This indicates that both residents with an underlying potential for bullying and residents with an underlying potential for victimisation will influence the social environment but will not have a huge impact on the physical environment, which is more likely to be shaped by other institutional factors. 

Similarly, while the black arrow leading from the outer ‘environmental box’ to the outer ‘nature and extent of bullying box’ indicates that both the physical and social environment will influence the overall nature and extent of bullying in the facility (including the nature and prevalence of bullying and victimisation, and the overlap between the two), the black arrows leading from the ‘potential for bullying box’ and the ‘potential for victimisation box’ are more specific and indicate that the former will increase the likelihood of bullying, whereas the latter will increase the likelihood of victimisation. The black arrow leading from the ‘interaction between bullies and victims box’ indicates that the interaction between bullies and victims (in the context of the special residential group dynamics) is likely to be particularly important in shaping the overlap between bullying and victimisation, while the black arrow leading from ‘the interaction between the environment and individual’ box indicates that the interaction between the environment and the individual will influence the overall nature and extent of bullying in the facility (i.e., bullying, victimisation and the overlap).

The MMB-RS argues that certain psychological and background characteristics of residents at intake will predispose some residents to become bullies and victims in their facility. Whether these potential bullies and victims become actual bullies and victims depends on the physical and social residential environment, as indicated by the grey arrow leading from the environmental factors to individual factors. The grey arrow leading from environmental to individual factors does not imply that the environment influences all individual factors equally. For instance, impulsivity might be genetically determined. Therefore, while the residential environment may contribute to amplifying an already existing impulsive tendency, it is unlikely to cause impulsivity. On the other hand, unsafe residential environments may, to a larger degree, contribute to creating the feelings of fear amongst residents or to developing attitudes approving of bullying, both of which may contribute to bullying behaviour. The physical environment includes the size of the facility, the size and number of living units per facility, the security level, the composition of residential groups, the level of crowding, material goods and services available to residents, furnishing, heating, the quality and the amount of food, and the staff to resident ratio [3,15,40]. Large, contained facilities with many living units and residents per unit, heterogeneous residential groups, staff shortages and limited material goods and services which provide stimulation and decrease boredom, are highly unlikely to portray a family-like environment and meet residents’ diverse needs. Rather, the model assumes that such facilities are more likely to reflect the residential environment in which the general well-being of residents may be seriously harmed. Such facilities may make residents feel that they need to take care of themselves and look for alternative ways to meet their needs, including bullying.

As indicated by the grey arrow in Figure 1 leading from the physical to the social environment, the model further suggests that the social environment is to some extent influenced by the physical environment and has an equally important impact on the behaviour of residents [3]. The social environment consists of the management and ideology of the facility, strategies used to maintain discipline, peer group dynamics and the residential peer subculture. The management and philosophy of the placement, as well as the strategies used to maintain discipline, arguably play an important role in determining the overall psychosocial climate of the institution [9,41]. The quality of the psychosocial climate will in turn influence the level of problem behaviours within the institution, including bullying [42].

Therefore, the model assumes that, if the philosophy of the residential placement is that bullying is an expected part of growing up, and if the attempts to deal with challenging behaviours of residents are merely based on rigid and authoritarian strategies that aim only to reduce problem behaviours but not to understand them, bullying is likely to be more prevalent. Poor leadership of the facility, where staff turnover is high, where staff are unclear about their roles, underpaid or at odds with each other, will further contribute to tensions in the facility. Indeed, there is evidence that problem behaviours of residents are less prevalent in establishments which are well managed, have a clear purpose, provide support to staff, rely on proactive strategies in maintaining discipline, and in which a caring and non-violent philosophy is clearly communicated to staff and residents from the ‘top’ of the establishment [9,41]. 

The MMB-RS further suggests that, apart from influencing bullying and victimisation, the physical environment of the residential facility, its ideology, management and child-rearing techniques will also have an impact on the *type* of residential peer subculture. This echoes prison-based research in terms of how the environment impacts [3]. Rigid and authoritarian strategies in dealing with challenging behaviour of residents are likely to be perceived as illegitimate and unfair by residents and consequently result in a poor relationship between residents and staff. The negative relationship with staff is in turn likely to result in a strong ‘us versus them’ mentality, leading to the creation of two different cultures, that of staff and that of residents. The stronger the ‘us versus them’ attitude, the more prison-like the physical environment, and the less caring the ideology of the facility, the more specifically defined the norms and values of the residential peer subculture are likely to become. As in prisons, these norms and values may lead to the creation of a residential code that may encourage bullying. If the main principles of the residential code are that residents should be tough, resist exploitation, avoid fraternising with staff and informing on their peers, and if violence is considered a legitimate way to stand up for oneself, bullying is likely to become ‘normalised’ [3,15]. 

Violations of the residential code may not only result in a variety of sanctions ranging from ostracising to physical violence, but deviation from and conforming to the code may also serve as the basis for determining the roles that residents undertake [33]. Such roles will in turn be closely related to group hierarchies often referred to as ‘pecking orders’ [3,5]. Indeed, as demonstrated above, there is evidence that residents of children’s homes who conform to the above-listed principles of the ‘residential code’ assume the role of a ‘top dog’ and are consequently positioned high within the residential hierarchies, while residents who inform on others are labelled ‘grassers’ and placed at the bottom of the ‘pecking order’ [5]. 

The level to which the pecking order is structured and dominance-based will largely depend on the type of the residential subculture and residential living arrangements. The more defiant the residential subculture and the larger and more heterogeneous the residential group, the more likely it is that the group hierarchy will be highly structured. The more structured the hierarchy of unbalanced power between the residents, the more likely the group dynamics are to become based on abusive relationships, with those in higher positions exploiting those in lower positions. Dominance-based group hierarchies and abusive group dynamics may be further enhanced if staff view peer hierarchies as a normal aspect of residential peer relationships, or if they rely on residents’ pecking orders as a mechanism of maintaining control. The practice of staff using ‘pecking orders’ in maintaining control over institutionalised individuals has been well documented in both early and more recent prison ethnography [33,43], as well as in research on young offenders’ institutions [44] and children’s residential care [5,13]. 

However, power relations and peer hierarchies are by no means static. Dramatic changes in peer group dynamics usually occur after a new admission or when residents who are towards the top of the hierarchy leave the placement [5,41,45]. These changes in peer group dynamics may lead to the longer-stay residents trying to protect their place in the group, often by means of bullying, intimidation and/or “initiation strategies” [5,13]. If new residents fail to resist adequately, they are automatically labelled as weak and placed at the bottom of the hierarchy. As in prisons, the avoidance of the vulnerable victim status may be an important part of a comfortable survival in the residential world [46] in which bullying is an adaptive behaviour or a ‘survival tool’ [3,44,47,48,49]. 

Highly structured residential groups, in which the avoidance of the vulnerable victim status is a priority, are likely to have complex exploitation systems that move beyond the mere division of bullies and victims. In firmly structured residential groups, not all victims will be positioned equally low and not all bullies will be positioned equally high in the ‘pecking order’. This will give some of the victims an opportunity to try to prevent their future victimisation and climb the hierarchy, either by bullying those lower down or by retaliating to less dominant bullies, who are not positioned at the top. Similarly, while still bullying those positioned below, some bullies may easily become victims of bullies higher up in the hierarchy. Therefore, the MBSS-RS argues for a more layered group structure and one that is dominance-based, lending itself to greater complexity between bullies and victims. As a result, the proportion of residents who are both bullies and victims (“bully/victims”) will consequently be large. It is also this understanding of a more layered approach to the bully–victim relationship, which is one of the most notable deviations from the MMBSS, which considered a more simple continuum-based understanding of these roles. 

How long “bully/victims” maintain their “bully/victim” status will again be largely dependent on the current composition of the residential group. Some “bully/victims” may become “pure bullies” as soon as more powerful bullies leave the placement, while some “bully/victims” may become “pure victims” when those positioned below them are discharged. Given that, in some types of residential placements, fluctuations of residents through intakes and discharges are extremely high, membership of the “bully/victim group” is likely to be highly unstable. The fact that Hanish and Guerra [50] found that membership of the “bully/victim” group amongst schoolchildren was only temporary, and that “bully/victims” in the Sekol and Farrington [31] and Sekol [7] studies were not in any way unique, further adds to this notion. The instability of the “bully/victim” status may also explain why certain personal characteristics seem to characterise both bullies and victims. In the studies by Sekol and Farrington [11,12], for instance, both bullies and victims were disagreeable, careless and neurotic, but these characteristics were more pronounced in bullies than in victims. It is possible, therefore, that residents with these personality traits are actually bullies, and that the residential peer culture and group dynamics occasionally make some of those bullies victims. This also fits with the MMBSS individual pathway route, which suggests a more trait-based understanding for those who bully [15]. However, the advantage of the MMB-RS lies in its dynamic nature, in that it suggests that the role of bully–victim may be more fluid; those who appear more frequently as ‘bullies’ may therefore reflect our understanding of ‘pure bullies’, as described in the prison-based models. 

Finally, the MMB-RS argues that while some environments are more likely to trigger personal predispositions for bullying and victimisation than others, some residents have stronger predispositions to become bullies or victims than others. The environmental clues are filtered through the pre-existing attributes of residents, their emotional states and their expectations [51]. Residents who have particularly strong predispositions to become bullies or victims will therefore interpret and react to their physical and social environment differently from their peers, who have weaker predispositions towards bullying or victimisation. For instance, residents who are disagreeable, careless, neurotic, hyperactive and impulsive may tend to interpret minor provocations as threatening, and consequently hold hostile attitudes towards others and respond aggressively even in environments not supportive of bullying. Similarly, residents who manifest symptoms of a larger syndrome of antisocial behaviour may feel more comfortable in engaging in bullying than their prosocial peers. Alternatively, residents who lack self-esteem, are depressed or anxious, have few friends, hold fatalistic attitudes about bullying and display submissiveness during peer disagreements, may become easy targets for bullying. 

Residents with a strong predisposition towards bullying or victimisation will not, however, contribute to the nature and extent of bullying in their placement only directly. That is, a great concentration of residents with a strong potential for bullying or victimisation is also likely to have a negative impact on the social environment of the residential placement, thus contributing to the creation of negative residential peer cultures, poor relationships with staff, strong peer hierarchies and the employment of reactive child-rearing techniques, all of which may lead to bullying and victimisation as described above. The impact of individual predispositions towards bullying and victimisation on the social environment is marked by the grey arrow in Figure 1, leading from individual characteristics to the social environment.

Another important aspect within the individual, and included in Figure 1, refers to the concept of fear. Fear is an emotional state that plays an important role in understanding bullying in closed social environments, and it is considered to be a motivating factor in explaining victim responses and the use of precautionary behaviours [30]. Unlike most other individual characteristics included in Figure 1, the MMB-RS assumes that fear is predominantly determined by the social environment of the residential placement. More precisely, in residential settings where there are strong peer hierarchies and where the prevalence of bullying is high, the (perceived or actual) risk of victimisation is also likely to be increased, which could in turn lead to generally high levels of fear amongst residents. This has been suggested in prison settings, where fear can lead to a flight response that can be immediate or delayed in victims [30]. The MMB-RS assumes that fear may lead to both bullying and victimisation. The way that fear may lead to bullying is twofold. It can either apply to residents who have not been bullied but who have witnessed others being bullied and who engage in bullying as a precaution to avoid their own victimisation, or it can apply to residents who have been bullied and who are aggressive either towards their bullies or other peers in order to prevent their future victimisation. This is captured as part of the Applied Fear Response Model [30] in prisons, where the complexity of the fear and bullying relationship is highlighted, with the constraints of the physical environment dictating and limiting the responses chosen by victims. 

Overall, the MMB-RS argues that the effects of environmental factors are inevitably mediated by the pre-existing attributes of residents. Given that these attributes may or may not include the potential for bullying and victimisation, a certain proportion of residents will most likely be involved in bullying and victimisation even in residential settings that are proactively disapproving of bullying. Similarly, some residents will remain uninvolved in bullying even in the residential environments most at risk. However, while the model argues that the effects of the residential environment are filtered through pre-existing individual characteristics, it does not neglect the potential influences that individual characteristics of residents may have on the social environment of the residential placement. This adds to the notion about dynamic, multilevel interrelationships between individual and environmental factors in contributing to bullying and victimisation in residential care. 

This also captures more recent conceptual considerations of bullying in secure settings, where an ecosystem framework has been applied. In the recently proposed Prison Bullying Ecosystem Framework [48], for example, there is emphasis on the dynamic interplay between the physical environment and the individuals who are housed. It captures the *external factors* known to be important in ecosystems—such as climate (social climate), changes to this climate, residual structures (e.g., social hierarchies), imported structures (e.g., imported characteristics and attitudes), materials and their movement (e.g., currency and access to goods) and the physical and cultural subculture of the environment. It also captures ecosystem *internal factors*—resource competition and shift, problematic groups and impact on dominance hierarchies, group composition and disruption to hierarchies. An ecosystem framework argues more for a process theory approach to understanding aggression in closed environments, which the MMB-RS is arguably beginning to reflect through its focus on dynamic processes and close attention to the interplay between the individual (i.e., the living component of an ecosystem) and the environment (i.e., the non-living component). Such elements become an important consideration for intervention approaches, since the value of a proposed model depends considerably on its applicability. 

### Testing the MMB-RS: Practical Considerations for a Programme of Intervention Research

While the MMB-RS may seem intuitively plausible, only empirically tested, refined versions of such a model could serve as the foundation for evidence-based interventions [48,52]. To test the proposed MMB-RS, an experimental programme of research could prove particularly useful, one that also incorporates an ecosystem processing approach. A non-experimental research design, which would test the effect of naturally occurring changes in the residential environment, as well as in individual characteristics of residents, on bullying and victimisation over time, does not seem to be ideal for residential care research. There are three reasons for this. First, in many residential facilities fluctuations of residents through intakes and discharges are high. Consequently, tracking naturally occurring changes in residents’ individual characteristics once they leave their facilities might be a difficult task. Second, given the stable nature of many individual factors (e.g., personality traits or empathy), expecting these factors to change noticeably over time, without deliberate attempts to modify them through intervention programmes, is unrealistic. Third, the effects of all three sets of factors (i.e., physical environment, social environment and individual characteristics) on bullying and victimisation can be tested more quickly in experimental than in non-experimental designs. Given the urgency to develop a theory of bullying in residential care, an experimental or quasi-experimental design seems more appropriate. Ireland et al. [39] applied such a method (quasi-experimental) to test the MMBSS in a high secure forensic hospital, where direct alterations to the physical and social environment were made. This was only applied to one, albeit sizeable, hospital and required full clinical and management adoption, which is not always achievable for researchers. Nevertheless, it demonstrated that such a method could be applied and that ensuring adherence to changes and quantifying this adherence was key. It was further evident from Ireland et al. [39] that statistical power was an unavoidable issue, if applied within a single site. Consequently, any true testing of a model such as the MMB-RS should include as many residential care facilities in one country as possible. 

The programme should start with an extensive data collection in each facility, including the most important variables measuring: (a) the physical environment of the facility (e.g., the size of the facility, the number of residents per bedroom, the number of staff per residential group, material goods and services available and so on); (b) the social environment of the facility (e.g., the ideology and the management of the facility, the expertise of staff, the psychosocial climate, the residential peer culture and so on); (c) the individual characteristics of residents (e.g., empathy, self-esteem, impulsivity, attitudes towards bullying, assertiveness and so on); and (d) the nature and extent of bullying and victimisation. While some measures could be assessed using pen and paper scales (e.g., psychosocial climate, empathy, self-esteem, impulsivity, bullying and victimisation), other measures might need to be assessed through observations, ethnography or focus groups (e.g., residential peer cultures and the ideology of the facility). 

After the first wave of data collection has been completed and baseline measures of the physical and social environment, individual characteristics and bullying and victimisation have been obtained, half of the facilities should be randomly allocated to receive programmes aimed at improving their physical and social environment (hereafter referred to as ‘experimental facilities’) and the other half should be control facilities. Residents from the experimental facilities, who were assessed as needing interventions aiming at their personal characteristics (e.g., empathy, impulsivity, attitudes approving of bullying, self-esteem etc.) should also receive the programmes that they need. 

The implementation of the programmes aiming at the three sets of factors (i.e., the physical environment, the social environment and individual factors) should be carried out with special care, with attention to the need to measure adherence and sufficient time given for programmes to begin having some effect (e.g., six to twelve months). Six to twelve months after the implementation of the programmes, manipulation checks should be conducted to establish whether the interventions have caused changes in the manipulated variables (i.e., the physical environment, the social environment and individual factors). If changes in the manipulated variables are found, it would then be crucial to establish whether variations in these variables had some effect on bullying and victimisation. It is also important to examine whether some elements of environmental or individual factors in the control facilities have changed, to rule out possible contamination effects. 

If after six to twelve months the experimental facilities demonstrate significant improvements in their physical and social environments, and reductions in bullying and victimisation compared to control facilities, this would provide good evidence that physical and social environments are related to bullying and victimisation, and that improving those aspects of residential living causes a reduction in bullying and victimisation. Similarly, if those residents from the experimental facilities who took part in programmes aiming at their personal characteristics (such as empathy, impulsivity or attitudes towards bullying) demonstrated significant desirable changes in these characteristics and decreases in their bullying behaviour compared to their matched peers from the control facilities, this would provide evidence that not only physical and social environments were related to bullying and victimisation, but that personal characteristics of residents also play an important role in shaping bullying in residential care.

## 3. Conclusions

Over the last decade, research on bullying in residential care has been increasing in scope and becoming more sophisticated. However, most of the existing research in this area has been conducted in a partial way, focusing either on a limited number of personal characteristics of residents or a limited number of elements of the prison environment. Consequently, no theory of bullying in residential care has been proposed. By adapting Ireland’s [15] MMBSS and integrating the results of the existing residential care bullying research, this paper is the first to propose a theoretical model of bullying in care. The proposed MMB-RS suggests that bullying in residential care is the result of dynamic and complex interactions between bullies and victims in the context of the special nature of the relatively closed physical and social residential care environment. In this way it also extends more recent applications of ecosystem frameworks to understanding bullying in secure settings [48], highlighting the contemporary nature of the model. The MMB-RS demonstrates its value to residential settings by (a) considering the social interactional components of bullying and victimisation in more detail, thus providing possible explanations of the large overlap between bullying and victimisation, as well as the ways that residential peer cultures, group dynamics and hierarchies may contribute to bullying; (b) including more specific individual variables, making a clear distinction between the psychological make-up of bullies and victims; and (c) adding some new environmental variables, which also map onto the more recent ecosystem approaches. 

Overall, the MMB-RS represents the first attempt to propose an applied theory of bullying in residential care. Clearly, it requires testing via empirical research that pays particular attention to examining *interactions* between bullies and victims, as well as interactions between bullies and victims and their residential social and physical environment. To test the MMB-RS, an experimental programme of research could be conducted. While such research may sound extremely time-consuming, complex and expensive, future studies of bullying in residential care need to attain a higher level of methodological quality and take into account all three sets of factors that may be associated with bullying and victimisation in residential care (i.e., physical, social and individual factors). Only empirically tested and refined theoretical models have convincing evidence-based applications to policy. Therefore, the proposed programme of research should, amongst other tasks, represent the most important task of future research.

## Data Availability

Not applicable.
